# Elevated serum substance P level as a predictive marker for moderately emetogenic chemotherapy‐induced nausea and vomiting: A prospective cohort study

**DOI:** 10.1002/cam4.3693

**Published:** 2020-12-27

**Authors:** Hyung Soon Park, Hye Sung Won, Ho Jung An, Sung Shim Cho, Hyun Ho Kim, Der Sheng Sun, Yoon Ho Ko, Byoung Yong Shim

**Affiliations:** ^1^ Department of Internal Medicine College of Medicine The Catholic University of Korea Seoul Korea; ^2^ Division of Medical Oncology Department of Internal Medicine St. Vincent's Hospital College of Medicine The Catholic University of Korea Suwon Korea; ^3^ Division of Medical Oncology Department of Internal Medicine Uijeongbu St. Mary's Hospital College of Medicine The Catholic University of Korea Uijeongbu Korea; ^4^ Division of Medical Oncology Department of Internal Medicine Eunpyeong St. Mary's Hospital College of Medicine The Catholic University of Korea Seoul Korea

**Keywords:** chemotherapy, nausea, predictive marker, substance P, vomiting

## Abstract

Chemotherapy‐induced nausea and vomiting (CINV) is an unbearable side effect. Identifying high emetic risk patients and providing more active antiemetics strategies are mandatory to improve the tolerability of chemotherapy. In this prospective cohort study, leptin, ghrelin, and substance P were measured at baseline, day 3, and day 14 during the first cycle of chemotherapy. Nausea and vomiting were measured each day for the first 4 days of the first cycle of chemotherapy. Eighty‐two patients were enrolled. Colorectal cancer (61%) and gastric cancer (35.4%) were common cancer types. All patients received moderate emetic risk chemotherapy. Forty‐five (54.9%) patients had nausea, and 15 (18.3%) patients experienced vomiting. In univariate analysis, a higher level of baseline substance P, which is a target of NK1‐RA (Neurokinin 1 receptor antagonist), was a significant predictive marker for chemotherapy‐induced nausea [odds ratio (OR): 2.6, 95% confidence interval (CI): 1.02–6.62, *p* = 0.046]. Regarding chemotherapy‐induced vomiting, patients with higher levels of substance P had a greater chance of vomiting [OR: 1.72, 95% CI: 0.49–5.99, *p* = 0.395] than those with lower levels of substance P. In patients receiving moderate emetic risk chemotherapy, active antiemetics, including NK1‐RA, could be considered for those with high levels of substance P.

## Graphical abstract

For patients with elevated substance P level, NK1‐RA (NK1 receptor antagonist, aprepitant) could be used, although these patients receive moderate emetic risk chemotherapy. This could improve the quality of life of patients receiving chemotherapy, and chemotherapy without dose reduction might have chance to improve treatment outcome for these patients.

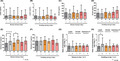

## 1 INTRODUCTION

According to the induction rate of chemotherapy‐induced nausea and vomiting (CINV), chemotherapy drugs can be classified as high, moderate, low, or minimal emetic risk.^1^ Guidelines recommend that patients treated with high emetic risk chemotherapy should receive a combination of neurokinin 1 (NK1) receptor antagonist (RA), serotonin (5‐HT) receptor antagonist, and dexamethasone as a premedication to prevent CINV.^2^ For moderate emetic risk chemotherapy, 5‐HT receptor antagonist and dexamethasone are recommended. Despite such premedication, approximately 30% of patients treated with moderate emetic risk chemotherapy experience significant nausea or vomiting.^3^, ^4^ CINV affects patient compliance with chemotherapy and leads to poor quality of life, an insufficient dose, and the delay or interruption of chemotherapy.^5^, ^6^ The prediction of patients at high risk for CINV can help ensure that they receive more intensive antiemetics treatment to reduce toxicity and improve compliance with chemotherapy.

However, risk factors for CINV have not yet been established. Although clinical factors such as young age, female sex, no alcohol history, morning sickness, and car sickness have been studied as high‐risk factors for CINV,^7^ biochemical markers are rarely studied. Neuropeptides and hormones are known to be related to appetite and vomiting, and they should be investigated. Leptin is primarily produced in adipose tissue. It is known to be an appetite decreasing hormone.^8^ The relationship between leptin and CINV remains unknown. However, pregnant women with high levels of leptin are known to have high risks for nausea and vomiting during pregnancy.^9^ Ghrelin, a gastrointestinal peptide hormone, was discovered in 1999.^10^ It is known to promote appetite.^10^ Patients with esophageal cancer show reduced levels of ghrelin after cisplatin chemotherapy.^11^ Substance P is known to be a key mediator of CINV. NK1‐RA has been developed to inhibit the interaction between substance P and the NK1 receptor.^12^

The objective of this study was to identify clinical and biochemical predictive markers for CINV. We also observed the dynamic changes in biochemical parameters after chemotherapy and analyzed them according to the presence or absence of CINV.

## 2 MATERIALS AND METHODS

### 2.1 Patients

In this prospective cohort study, patients with colorectal, gastric, pancreatic, or small bowel cancer who received moderate emetic risk chemotherapy from March 2014 to August 2015 at St. Vincent's Hospital and Uijeongbu St. Mary's Hospital were enrolled. Patients who had brain metastasis or intestinal obstruction were excluded. All patients received granisetron patch on the day before starting the first cycle of chemotherapy. Dexamethasone and dopamine antagonist were used as rescue drugs. The following data were documented for all subjects: age, sex, weight loss within 6 months, BMI (body mass index), history of smoking, drinking, morning sickness and car sickness, anxiety regarding chemotherapy, tumor‐node‐metastasis (TNM) stage, previous chemotherapy, chemotherapy regimen, diagnosis (primary site), and comorbidity. As biochemical predictive markers for CINV, leptin, ghrelin, and substance P were measured using serum collected during chemotherapy. Nausea and vomiting were recorded for patients on days 1, 2, 3, and 4 of the first cycle of chemotherapy; severity was measured using VAS (visual analog scale) scored from 0 to 100 points. This prospective cohort study was approved by the Institutional Review Board (IRB) of St. Vincent's Hospital and the IRB of Uijeongbu St. Mary's Hospital (XC13TIMI0063V).

### 2.2 Measurement of hormones and neuropeptides

Serum was collected to measure leptin, ghrelin, and substance P levels to determine their clinical roles in CINV. Blood collection was performed three times: in the morning before chemotherapy (day 1, baseline), 2 days after chemotherapy (day 3), and 13 days after chemotherapy (day 14). Serum was isolated from 10 ml of peripheral blood collected in an SST (serum‐separating) tube. Serum for leptin measurement was moved to a conical tube. AEBSF (4‐(2‐aminoethyl)benzenesulfonyl fluoride) hydrochloride and aprotinin (Sigma‐Aldrich, St. Louis, MO, USA)‐treated conical tubes were used for ghrelin and substance P measurements, respectively. These conical tubes were incubated at room temperature for 30 minutes, and then, centrifuged at 3,000 rpm for 15 minutes at 4°C. The supernatant was collected and stored at −80°C until analysis.

Serum supernatants were used to measure the levels of circulating leptin, ghrelin, and substance P with commercially available enzyme‐linked immunosorbent assay (ELISA) kits (Millipore, Billerica, MA; R&D Systems, Abingdon, UK) following the manufacturers’ protocols. Samples were measured in triplicate, and the mean value was used as the final concentration.

### 2.3 Statistics

Levels of hormones and neuropeptides are presented as the median ±interquartile range (IQR). The results were compared using the Mann–Whitney *U*‐test. Hormones and neuropeptides were initially recorded as continuous variables, and then, later dichotomized according to ROC (receiver operating characteristic) curve analysis.^13^ Sensitivity and specificity for the prediction of nausea and vomiting were plotted to generate the ROC curve. Nausea and vomiting were evaluated according to the NCI‐CTC (National Cancer Institute‐Common Toxicity Criteria); severity was defined with a 100‐mm visual analog scale (VAS). Correlation testing was performed using Pearson's correlation coefficient, with a value of >0.7 indicating good correlation.^14^ Logistic regression was used to evaluate associations between clinico‐biochemical factors and CINV. Statistical significance was considered at *p* < 0.05. PASW Statistics 18.0 (SPSS Inc. Chicago, IL, USA) was used for all statistical analyses.

## 3 RESULTS

### 3.1 Patient characteristics

Eighty‐two patients were enrolled. Their median age was 61 years (Table 1). All patients received moderate emetic risk chemotherapy. FOLFIRI (39, 47.6%) was the most frequently used chemotherapy regimen, followed by the XELOX (23, 28%) and FOLFOX (20, 24.4%) regimens. Regarding primary cancer type, colorectal cancer (50, 61%) and gastric cancer (29, 35.4%) were the most common cancers.

**TABLE 1:** Baseline characteristics of the subjects

Characteristics	Number	%
Age, years (median, range)	61	(40–84)
Sex (n, %)		
Male	57	69.5
Female	25	30.5
Weight loss (within 6 months)		
No	25	30.5
Yes	57	69.5
BMI (median, range), kg/m^2^	22.0	(14.4–54.1)
Estimated GFR (median, range), ml/min/1.73 m^2^ (by MDRD)	83	(39.6–179.7)
Smoking (n, %)		
Never smoker	31	37.8
Ex‐smoker	39	47.6
Current smoker	12	14.6
Drinking (n, %)		
No	49	59.8
Yes	28	34.1
Morning sickness (n, %)		
No	7	8.5
Yes	12	14.6
Car sickness (n, %)		
No	62	75.6
Yes	20	24.4
Anxiety (n, %)		
No	55	67.1
Yes	27	32.9
TNM stage (n, %)		
2	4	4.9
3	17	20.7
4	61	74.4
Previous chemotherapy (n, %)		
No	68	82.9
Yes	14	17.1
Chemotherapy regimen (n, %)		
FOLFIRI	39	47.6
FOLFOX	20	24.4
XELOX	23	28
Diagnosis (n, %)		
Colorectal cancer	50	61
Gastric cancer	29	35.4
Pancreatic cancer	1	1.2
Small bowel cancer	2	2.4
Comorbidity (n, %)		
DM	6	7.3
Hypertension	12	14.6
DM & Hypertension	9	11
BPH	1	1.2
CVD	2	2.4

Abbreviations: BMI, body mass index; BPH, benign prostate hyperplasia; CVD, cardiovascular disease; DM, diabetes mellitus; FOLFIRI, 5‐FU leucovorin irinotecan; FOLFOX, 5‐FU leucovorin oxaliplatin; GFR, glomerular filtration rate; MDRD, modification of diet in renal disease; N, number; XELOX, capecitabine, oxaliplatin.

**FIGURE 1:**
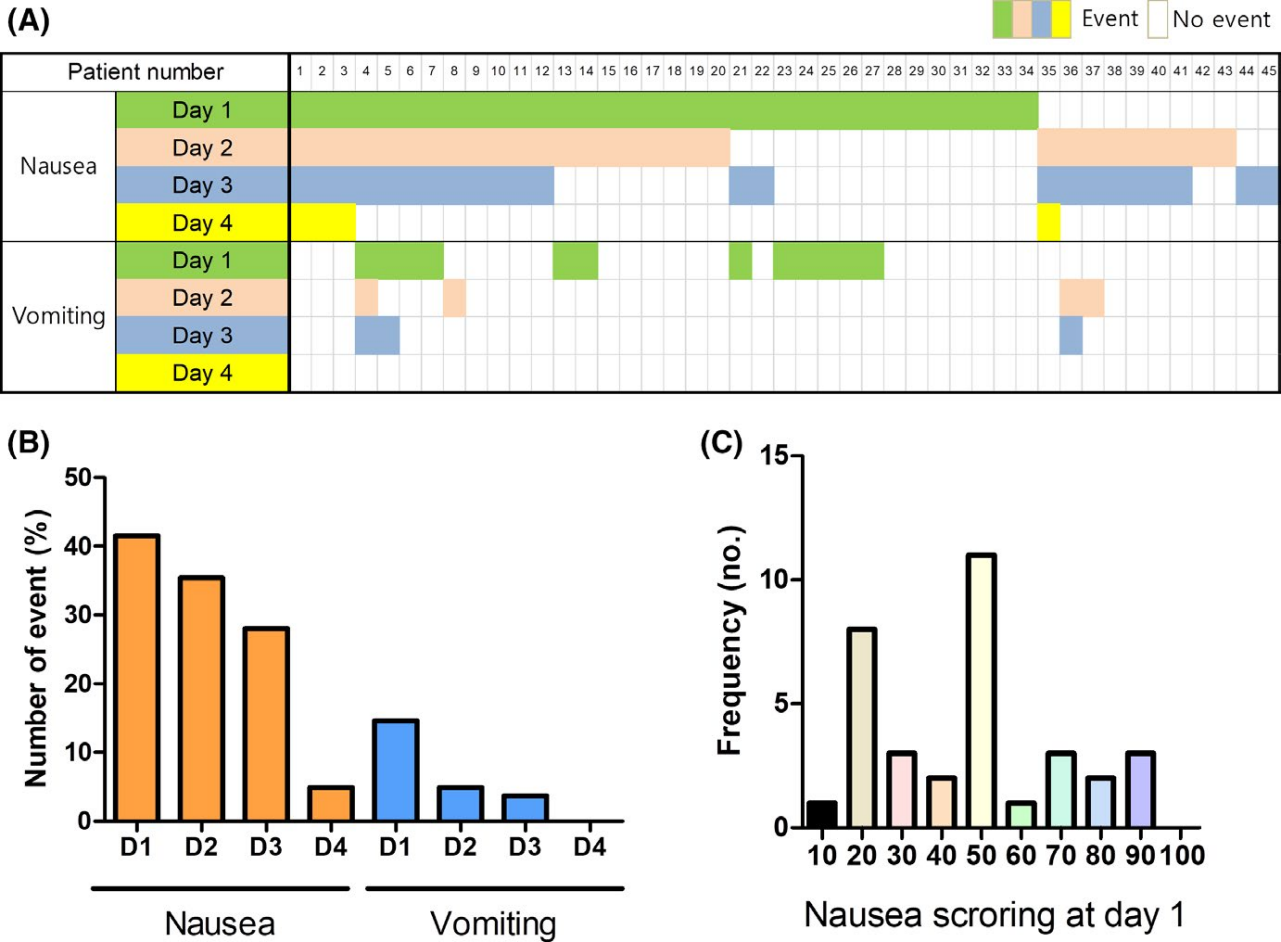
Pattern of chemotherapy‐induced nausea and vomiting in patients receiving moderate emetic risk chemotherapy. A, Nausea and vomiting according to days after chemotherapy. B, Forty‐two percent of patients experienced nausea on day 1, and 14.6% of patients had vomiting on day 1 after chemotherapy. C, Regarding the severity of nausea, 20 patients experienced nausea with a VAS score of 50 or more. The Mann–Whitney *U*‐test was used to compare continuous values

Nausea developed in 45 (54.9%) patients during the 4 days after chemotherapy (Figure 1A). Many (n = 34, 41.5%) patients developed nausea within 24 hours. Fifteen (18.3%) patients had vomiting during the 4 days after chemotherapy. Of them, 12 experienced vomiting within the first 24 hours (Figure 1B). Severity of nausea at day 1 was evaluated. Twenty patients had nausea with VAS scores of 50 or more, and five patients experienced more severe nausea with VAS scores of 80 or more (Figure 1C).

### 3.2 Analysis of biochemical factors

The baseline median levels of leptin, ghrelin, and substance P were 1.5 ng/ml, 198.3 pg/ml, and 301.2 pg/ml, respectively (Table 2). There were no significant correlations among the biochemical markers at baseline (Figure S1). The dynamics of the biochemical markers during chemotherapy were also evaluated. There were no significant differences in biochemical markers among the different time points (Figure S2).

**TABLE 2:** Levels of leptin, substance P, and ghrelin

Characteristics	Median (IQR)
Leptin, ng/ml	
Day 1	1.5 (0.6–4.9)
Day 3	1.4 (0.6–5.1)
Day 14	1.5 (0.6–4.2)
Ghrelin, pg/ml	
Day 1	198.3 (61.7–353.4)
Day 3	203.4 (90.5–336.7)
Day 14	239.7 (136.9–428.2)
Substance P, pg/ml	
Day 1	301.2 (179.2–420.5)
Day 3	320.8 (204.6–423.9)
Day 14	311.6 (196.3–434.3)

Abbreviation: IQR interquartile range.

**FIGURE 2:**
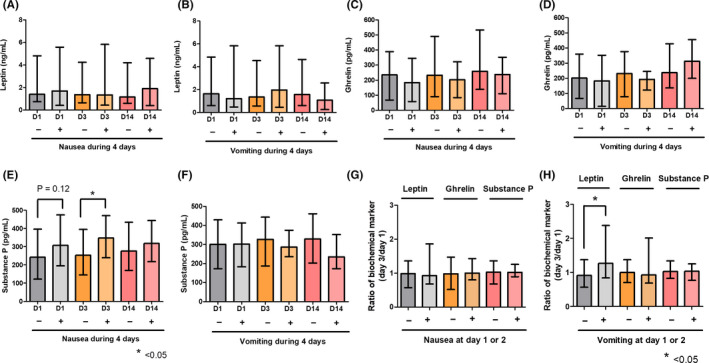
Levels of biochemical markers according to the presence of CINV. A and B, Leptin level according to the presence of nausea or vomiting during the 4 days after chemotherapy. C and D, Ghrelin level according to the presence of nausea or vomiting during the 4 days after chemotherapy. E and F, Substance P level according to the presence of nausea or vomiting during the 4 days after chemotherapy. Patients with nausea had higher levels of substance P on day 1 (baseline) and day 3 after chemotherapy. G and H, Changes in leptin, ghrelin, and substance P levels after chemotherapy according to the presence of nausea or vomiting on day 1 or day 2. Patients with vomiting had significantly increased leptin levels. The Mann–Whitney *U*‐test was used to compare continuous values

Levels of leptin, ghrelin, and substance P were compared between patients with and without nausea/vomiting during the 4 days after chemotherapy. Leptin and ghrelin levels were not significantly different between these groups (Figure 2A–D). Patients with nausea had higher levels of substance P than those without nausea at baseline and on day 3 (Figure 2E). However, there were no significant differences in the levels of biochemical markers between patients with and without vomiting at any time point (Figure 2F). Changes in biochemical markers according to the presence of nausea/vomiting were also analyzed (Figure 2G–H). Patients with vomiting events on day 1 or day 2 had significantly increased leptin levels at day 3 compared to baseline.

### 3.3 Predictive factors for CINV

The best cutoff points for leptin, ghrelin, and substance P by ROC curve analysis for the prediction of chemotherapy‐induced nausea (CIN) were 5.8 ng/ml, 201.6 pg/ml, and 222.6 pg/ml, respectively. In univariate analysis, a higher level of baseline substance P was the only significant predictive marker for chemotherapy‐induced nausea (*p* = 0.046) (Table 3). Regarding chemotherapy‐induced vomiting (CIV), patients with high levels of substance P had a higher chance of vomiting, although this difference did not reach statistical significance (*p* = 0.395). Chemotherapy regimen and cancer type were significant predictive markers for chemotherapy‐induced vomiting. In multivariate analysis, baseline substance P level was an independent predictive marker for chemotherapy‐induced nausea (*p* = 0.032). In addition, patients with higher levels of leptin had more events of chemotherapy‐induced nausea (*p* = 0.043) than patients with lower levels. As an independent predictive marker for chemotherapy‐induced vomiting, patients receiving the FOLFOX regimen had a greater chance of vomiting than patients receiving FOLFIRI. In subgroup analysis by chemotherapy regimen and diagnosis, a high level of substance P was significantly associated with chemotherapy‐induced nausea in gastric cancer and patients who received the XELOX regimen. However, there was no significant association between substance P level and chemotherapy‐induced vomiting in any subgroup (Table S1).

**TABLE 3:** Univariate and multivariate analyses for CINV

Characteristics	No	Nausea						Vomiting					
		Univariate			Multivariate			Univariate			Multivariate		
		OR	95% CI	*P*‐value	OR	95% CI	*P*‐value	OR	95% CI	*P*‐value	OR	95% CI	*P*‐value
Age, years													
≤61	45	1.00		0.757				1.00		0.894			
>61	37	1.15	0.48–2.76					1.08	0.35–3.32				
Sex													
Male	57	1.00		0.118				1.00		0.791			
Female	25	2.20	0.82–5.91					1.18	0.36–3.88				
Weight loss (within 6 months)													
No	25	1.00		0.408				1.00		0.379			
Yes	57	1.49	0.58–3.83					0.59	0.19–1.90				
BMI, kg/m^2^													
<20	25	1.00		0.538				1.00		0.379			
≥20	57	0.74	0.29–1.92					0.59	0.19–1.90				
Estimated GFR, ml/min/1.73 m2 (by MDRD)													
≥60	69	1.00		0.094	1.00		0.059	1.00		0.628			
<60	13	3.24	0.82–12.79		4.04	0.95–17.17		1.43	0.34–5.97				
Smoking													
Never smoker	31	1.00		0.774				1.00		0.742			
Ex‐smoker	39	1.35	0.52–3.49					0.76	0.22–2.63				
Current smoker	12	0.94	0.25–3.56					1.39	0.29–6.75				
Drinking													
No	49	1.00		0.897				1.00		0.955			
Yes	28	0.94	0.37–2.39					0.97	0.29–3.23				
Morning sickness													
No	7	1.00		0.679				1.00		0.585			
Yes	12	1.50	0.22–10.22					2.00	0.17–24.07				
Car sickness													
No	62	1.00		0.597				1.00		0.820			
Yes	20	1.32	0.47–3.67					1.16	0.32–4.15				
Anxiety													
No	55	1.00		0.577				1.00		0.216			
Yes	27	1.30	0.51–3.31					2.06	0.66–6.44				
TNM stage													
2	4	1.00		0.738				1.00		0.939			
3	17	0.89	0.10–7.86					0.64	0.05–8.52				
4	61	1.35	0.18–10.19					0.66	0.06–6.96				
Previous chemotherapy													
No	68	1.00		0.688				1.00		0.671			
Yes	14	0.79	0.25–2.50					0.71	0.14–3.54				
Chemotherapy regimen													
FOLFIRI	39	1.00		0.130				1.00		0.083	1.00		0.041
FOLFOX	20	3.02	0.96–9.51					4.71	1.18–18.80		16.51	1.72–158.89	
XELOX	23	2.01	0.71–5.75					1.84	0.41–8.21		4.28	0.32–56.80	
Diagnosis													
Colorectal cancer	50	1.00		0.315				1.00		0.067	1.00		0.047
Gastric cancer	29	1.90	0.74–4.89					4.05	1.20–13.63		1.51	0.24–9.70	
Others^a^	3	0.50	0.04–5.87					4.50	0.34–58.92		0.13	0.01–1.95	
Leptin, ng/ml													
Low (<5.8)	64	1.00		0.076	1.00		0.043	1.00		0.333			
High (≥5.8)	14	3.44	0.88–13.52		4.37	1.05–18.22		1.93	0.51–7.28				
Ghrelin, pg/ml													
Low (<201.6)	41	1.00		0.187				1.00		0.454			
High (≥201.6)	39	0.55	0.22–1.34					0.65	0.21–2.03				
Substance P, pg/ml													
Low (<222.6)	29	1.00		0.046	1.00		0.032	1.00		0.395			
High (≥222.6)	51	2.60	1.02–6.62		3.09	1.10–8.69		1.72	0.49–5.99				

Abbreviations: BMI, body mass index; CI, confidence interval; FOLFIRI, 5‐FU, leucovorin, irinotecan; FOLFOX, 5‐FU, leucovorin, oxaliplatin; GFR, glomerular filtration rate; MDRD, modification of diet in renal disease; No, number; OR, odds ratio; XELOX, capecitabine, oxaliplatin.

^a^ Others include pancreatic cancer, bladder cancer, and small bowel cancer.

## 4 DISCUSSION

Nausea and vomiting are common and critical side effects for patients receiving chemotherapy. Several kinds of clinical factors have been reported to predict CINV, including young age and female sex.^7^ However, biochemical markers have not yet been well studied. This study showed that a high level of baseline substance P was associated with CINV. This finding could help high emetic risk patients receive more intensive antiemetics treatment, such as NK1‐RA that targets substance P, prior to chemotherapy. This could improve the quality of life of patients receiving chemotherapy, and chemotherapy without dose reduction might improve treatment outcome for these patients.

Multiple neurotransmitters, including dopamine, serotonin (5‐HT), and substance P, are known to be associated with the pathophysiology of CINV.^15^ Antiemetics that target these neurotransmitters have been developed for the prevention and symptom improvement of CINV. Although the role of dopamine is less clear, dopamine receptor antagonists are commonly used antiemetics and likely to have anti‐dyspeptic effects.^16^ In general, serotonin plays a major role in acute CINV but a lesser role in delayed CINV.^17^ With the advent of serotonin antagonists, prophylaxis for CINV represents a major step toward better patient tolerance and adherence to chemotherapy. However, approximately 60% of patients receiving moderately emetogenic chemotherapy experienced nausea during the acute and delayed phases (0–120 hours), even though a serotonin antagonist was used to prevent CINV.^3^ Despite the premedication, clinically significant nausea (VAS scale ≥25 mm) and vomiting were observed in 33% and 35%, respectively.^3^

Many studies have been conducted to identify clinical predictive factors for CINV^18^, ^19^ and several high‐risk factors, including young age, female sex, poor performance status, prior chemotherapy exposure, and no prior use of alcohol, have been reported. This study evaluated these clinical factors in patients receiving moderate emetic risk chemotherapy in a prospective manner. However, young age (less than 62 years), no prior use of alcohol, morning sickness, and car sickness were not associated with CINV. Although female sex, decreased renal function and the FOLFOX chemotherapy regimen tended to be high‐risk factors for chemotherapy‐induced nausea in the current study, they failed to reach statistical significance. The chemotherapy regimen and type of cancer, especially gastric cancer, were significant high‐risk factors for chemotherapy‐induced vomiting.

Patients with high levels of leptin or low levels of ghrelin tended to have increased risk of CINV. Leptin and ghrelin have opposite effects on energy homeostasis. Leptin induces suppression of food intake, while ghrelin stimulates appetite. Consistent with this principle, changes in leptin and ghrelin levels after chemotherapy moved in opposite directions. However, leptin does not regulate ghrelin levels, and they function independently of each other in the control of energy homeostasis.^20^ In our study, patients with vomiting during the 2 days after chemotherapy had significant increases in leptin levels at day 3 compared to day 1 than patients without vomiting. Vomiting is one of the causes of poor appetite. This might be related to increased leptin, which is known as an anti‐appetite hormone, and our data were consistent with this explanation. However, this hypothesis should be further evaluated in more studies dealing with chemotherapy. High substance P levels before chemotherapy significantly affected CINV development in the study. Previous studies have focused on increased substance P levels after cytotoxic chemotherapy.^21^ Similar to serotonin, substance P release is known to be mediated by chemotherapy. It appears to bind largely to NK1 receptors that are centrally located.^22^ Takahashi et al^21^ reported that substance P levels were significantly increased on days 2–4 after chemotherapy in patients with delayed nausea or vomiting in subgroup analysis, supporting the possibility that increased substance P might be involved in the pathogenesis of CINV.

However, our study showed that substance P levels at day 3 after chemotherapy were not increased compared to baseline. This finding was consistently observed regardless of CINV development. In contrast, high substance P levels before chemotherapy were observed in patients with CINV. Substance P levels in patients with nausea were also higher on day 3 and day 14 than those in patients without nausea. These findings suggest that the underlying substance P level is more important to the development of CINV than its incremental change after chemotherapy. Higa et al^23^ have also reported that baseline substance P levels were threefold higher in patients with emesis than in those without emesis. This finding was consistently observed in expanded patients of their study group.^24^ Darmani et al^25^ reported that overexpression of the NK1 receptor is induced after chemotherapy. This mechanism could explain how higher baseline substance P levels affect CINV development. In our study, a possible explanation for the lack of an increase in substance P levels after chemotherapy is that moderately emetic chemotherapy was used. In the study conducted by Higa et al., increased substance P was observed in patients treated with high‐dose cisplatin (≥75 mg/m^2^) but not in patients treated with low‐dose cisplatin.

In conclusion, this is the first prospective cohort study showing that baseline substance P level is a significant predictive marker for nausea in patients treated with moderate emetic chemotherapy. For patients with elevated substance P levels, NK1 receptor antagonists could be used, even though these patients receive moderate emetic risk chemotherapy. The findings of this study need to be validated with further studies. Furthermore, based on future validation studies, intervention studies according to substance P levels might be performed in the future.

## 5 ETHNICITY

This prospective cohort study was approved by the Institutional review board (IRB) of St. Vincent's hospital and the IRB of Uijeongbu St. Mary's hospital (XC13TIMI0063V). Written informed consent was obtained from all the patients recruited prior to commencement of this study.

## CONFLICT OF INTEREST

None declared.

## AUTHOR CONTRIBUTIONS

Hyung Soon Park: data analysis, writing, editing. Hye Sung Won: design, data collection, assembly of the data. Ho Jung An: data collection, review. Sung Shim Cho: supervision, review. Hyun Ho Kim: supervision, review. Der Sheng Sun: data collection, supervision. Yoon Ho Ko: data collection, interpretation, review. Byoung Yong Shim: conception, design, interpretation, review. All authors approved the final manuscript.

## Supporting information

Fig S1

Fig S2

Table S1

## Data Availability

Data are available upon reasonable request and it can be obtained from the corresponding author, BYS.

## References

[1] Roila F , Molassiotis A , Herrstedt J , et al. 2016 MASCC and ESMO guideline update for the prevention of chemotherapy‐ and radiotherapy‐induced nausea and vomiting and of nausea and vomiting in advanced cancer patients. Ann Oncol. 2016;27:v119‐v133. 27664248 10.1093/annonc/mdw270

[2] Hesketh PJ , Kris MG , Basch E , et al. Antiemetics: American Society of Clinical Oncology Clinical Practice Guideline Update. J Clin Oncol. 2017;35:3240‐3261. 28759346 10.1200/JCO.2017.74.4789

[3] Schwartzberg LS , Modiano MR , Rapoport BL , et al. Safety and efficacy of rolapitant for prevention of chemotherapy‐induced nausea and vomiting after administration of moderately emetogenic chemotherapy or anthracycline and cyclophosphamide regimens in patients with cancer: a randomised, active‐controlled, double‐blind, phase 3 trial. Lancet Oncol. 2015;16:1071‐1078. 26272768 10.1016/S1470-2045(15)00034-0

[4] Escobar Y , Cajaraville G , Virizuela JA , et al. Incidence of chemotherapy‐induced nausea and vomiting with moderately emetogenic chemotherapy: ADVICE (Actual Data of Vomiting Incidence by Chemotherapy Evaluation) study. Support Care Cancer. 2015;23:2833‐2840. 26081597 10.1007/s00520-015-2809-3 PMC4519584

[5] Aapro M . CINV: still troubling patients after all these years. Support Care Cancer. 2018;26:5‐9. 29556808 10.1007/s00520-018-4131-3 PMC5876280

[6] Rusthoven JJ , Osoba D , Butts CA , et al. The impact of postchemotherapy nausea and vomiting on quality of life after moderately emetogenic chemotherapy. Support Care Cancer. 1998;6:389‐395. 9695208 10.1007/s005200050182

[7] Dranitsaris G , Molassiotis A , Clemons M , et al. The development of a prediction tool to identify cancer patients at high risk for chemotherapy‐induced nausea and vomiting. Ann Oncol. 2017;28:1260‐1267. 28398530 10.1093/annonc/mdx100 PMC5452068

[8] Ahima RS , Prabakaran D , Mantzoros C , et al. Role of leptin in the neuroendocrine response to fasting. Nature. 1996;382:250‐252. 8717038 10.1038/382250a0

[9] Lee NM , Saha S . Nausea and vomiting of pregnancy. Gastroenterol Clin North Am. 2011;40(2):309‐334. 21601782 10.1016/j.gtc.2011.03.009 PMC3676933

[10] Kojima M , Hosoda H , Date Y , et al. Ghrelin is a growth‐hormone‐releasing acylated peptide from stomach. Nature. 1999;402:656‐660. 10604470 10.1038/45230

[11] Hiura Y , Takiguchi S , Yamamoto K , et al. Fall in plasma ghrelin concentrations after cisplatin‐based chemotherapy in esophageal cancer patients. Int J Clin Oncol. 2012;17:316‐323. 21773688 10.1007/s10147-011-0289-0

[12] Hesketh PJ , Grunberg SM , Gralla RJ , et al. The oral neurokinin‐1 antagonist aprepitant for the prevention of chemotherapy‐induced nausea and vomiting: a multinational, randomized, double‐blind, placebo‐controlled trial in patients receiving high‐dose cisplatin–the Aprepitant Protocol 052 Study Group. J Clin Oncol. 2003;21:4112‐4119. 14559886 10.1200/JCO.2003.01.095

[13] Hanley JA . Receiver operating characteristic (ROC) methodology: the state of the art. Crit Rev Diagn Imaging. 1989;29:307‐335. 2667567

[14] Mukaka MM . Statistics corner: a guide to appropriate use of correlation coefficient in medical research. Malawi Med J. 2012;24:69‐71. 23638278 PMC3576830

[15] Navari RM , Aapro M . Antiemetic prophylaxis for chemotherapy‐induced nausea and vomiting. N Engl J Med. 2016;374:1356‐1367. 27050207 10.1056/NEJMra1515442

[16] Janelsins MC , Tejani MA , Kamen C , et al. Current pharmacotherapy for chemotherapy‐induced nausea and vomiting in cancer patients. Expert Opin Pharmacother. 2013;14:757‐766. 23496347 10.1517/14656566.2013.776541 PMC3938333

[17] del Giglio A , Soares HP , Caparroz C , et al. Granisetron is equivalent to ondansetron for prophylaxis of chemotherapy‐induced nausea and vomiting: results of a meta‐analysis of randomized controlled trials. Cancer. 2000;89:2301‐2308. 11147601 10.1002/1097-0142(20001201)89:11<2301::aid-cncr19>3.0.co;2-6

[18] Pollera CF , Giannarelli D . Prognostic factors influencing cisplatin‐induced emesis. Definition and validation of a predictive logistic model. Cancer. 1989;64:1117‐1122. 2667749 10.1002/1097-0142(19890901)64:5<1117::aid-cncr2820640525>3.0.co;2-r

[19] Hesketh PJ , Aapro M , Street JC , et al. Evaluation of risk factors predictive of nausea and vomiting with current standard‐of‐care antiemetic treatment: analysis of two phase III trials of aprepitant in patients receiving cisplatin‐based chemotherapy. Support Care Cancer. 2010;18:1171‐1177. 19756774 10.1007/s00520-009-0737-9

[20] Klok MD , Jakobsdottir S , Drent ML . The role of leptin and ghrelin in the regulation of food intake and body weight in humans: a review. Obes Rev. 2007;8:21‐34. 17212793 10.1111/j.1467-789X.2006.00270.x

[21] Takahashi T , Nakamura Y , Tsuya A , et al. Pharmacokinetics of aprepitant and dexamethasone after administration of chemotherapeutic agents and effects of plasma substance P concentration on chemotherapy‐induced nausea and vomiting in Japanese cancer patients. Cancer Chemother Pharmacol. 2011;68:653‐659. 21125277 10.1007/s00280-010-1519-2 PMC3162145

[22] Diemunsch P , Grelot L . Potential of substance P antagonists as antiemetics. Drugs. 2000;60:533‐546. 11030465 10.2165/00003495-200060030-00002

[23] Higa GM , Auber ML , Altaha R , et al. 5‐Hydroxyindoleacetic acid and substance P profiles in patients receiving emetogenic chemotherapy. J Oncol Pharm Pract. 2006;12:201‐209. 17156592 10.1177/1078155206072080

[24] Higa GM , Auber ML , Hobbs G . Identification of a novel marker associated with risk for delayed chemotherapy‐induced vomiting. Support Care Cancer. 2012;20:2803‐2809. 22350597 10.1007/s00520-012-1402-2

[25] Darmani NA , Dey D , Chebolu S , et al. Cisplatin causes over‐expression of tachykinin NK(1) receptors and increases ERK1/2‐ and PKA‐ phosphorylation during peak immediate‐ and delayed‐phase emesis in the least shrew (Cryptotis parva) brainstem. Eur J Pharmacol. 2013;698:161‐169. 23001014 10.1016/j.ejphar.2012.09.008

