# Supplementary figures and images for: Elevated serum substance P level as a predictive marker for moderately emetogenic chemotherapy‐induced nausea and vomiting: A prospective cohort study
Source: Cancer Med. 2020 Dec 27;10(3):1057–65. doi: 10.1002/cam4.3693 (PMC7897939; doi:10.1002/cam4.3693)

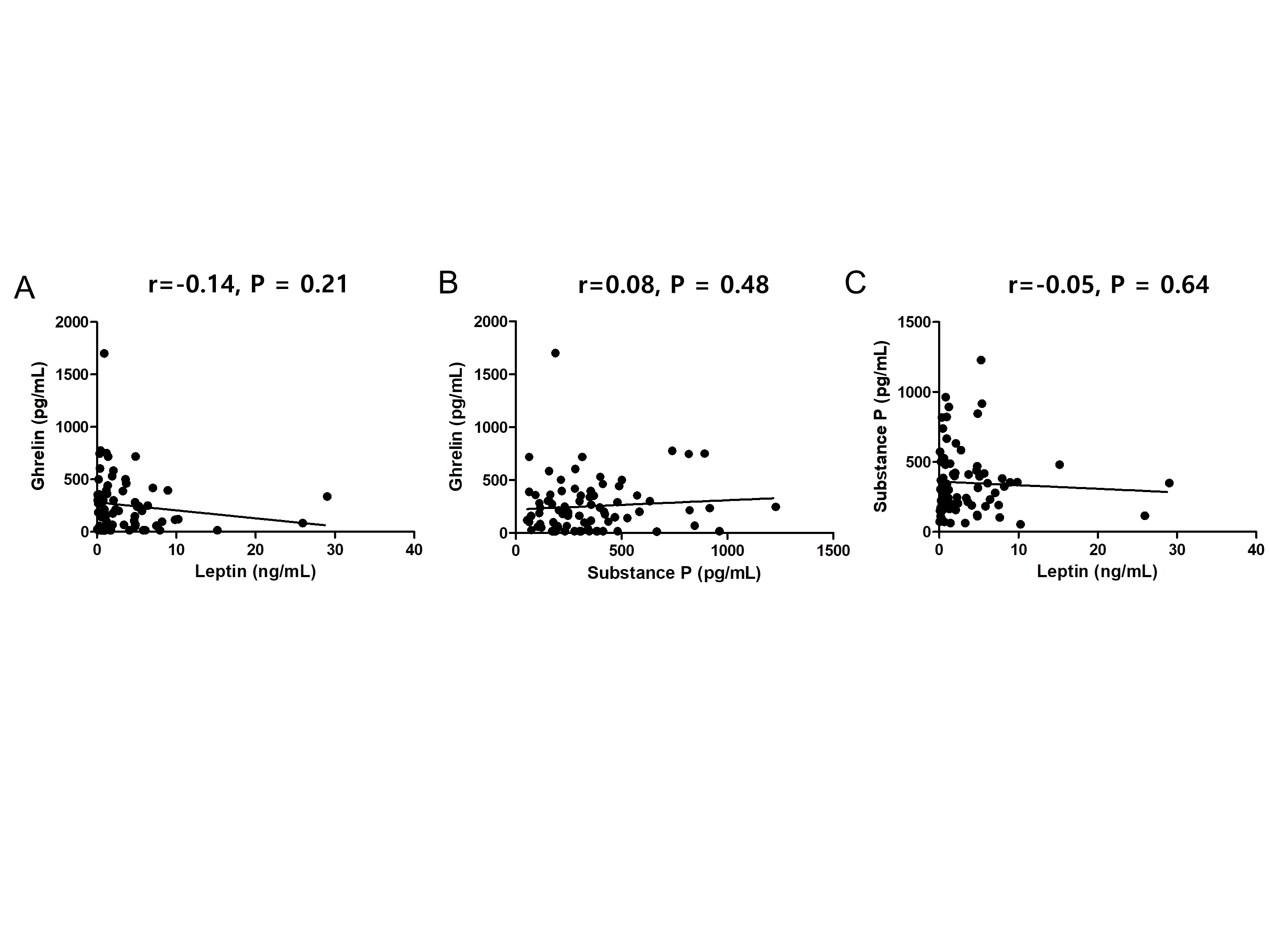

Supplement: Supplementary file 1 — Fig S1 [file CAM4-10-1057-s001.TIF]

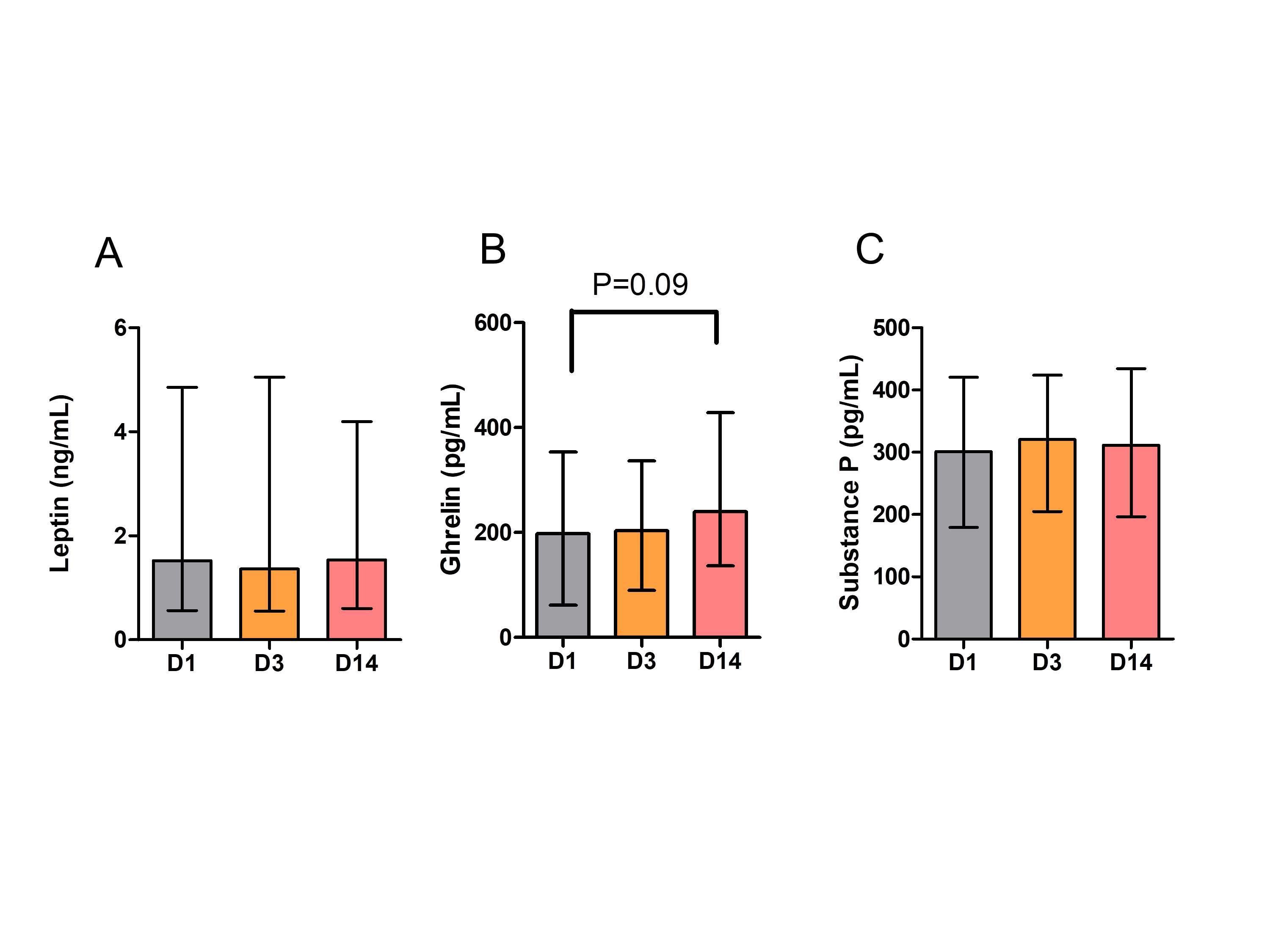

Supplement: Supplementary file 2 — Fig S2 [file CAM4-10-1057-s002.TIF]
