# Supplementary material for: Elevated serum substance P level as a predictive marker for moderately emetogenic chemotherapy‐induced nausea and vomiting: A prospective cohort study
Source: Cancer Med. 2020 Dec 27;10(3):1057–65. doi: 10.1002/cam4.3693 (PMC7897939; doi:10.1002/cam4.3693)
Supplement: Supplementary file 3 — Table S1 [file CAM4-10-1057-s003.docx]

Supporting information Table 1. Univariate analysis for chemotherapy induced nausea and vomiting in subgroup

| Characteristics |  |  | Nausea | | | | Vomiting | | | |
| --- | --- | --- | --- | --- | --- | --- | --- | --- | --- | --- |
|  |  |  | OR | 95% CI | | P-value | OR | 95% CI | | P-value |
| Diagnosis |  |  |  |  |  |  |  |  |  |  |
|  | Colon cancer |  |  |  |  |  |  |  |  |  |
|  | Substance P | Low | 1.00 |  |  | 0.758 | 1.00 |  |  | 0.610 |
|  |  | High | 1.21 | 0.36 | 4.07 |  | 0.61 | 0.09 | 4.08 |  |
|  | Gastric cancer |  |  |  |  |  |  |  |  |  |
|  | Substance P | Low | 1.00 |  |  | 0.012 | 1.00 |  |  | 0.113 |
|  |  | High | 19.60 | 1.91 | 201.62 |  | 4.38 | 0.71 | 27.16 |  |
| Regimen |  |  |  |  |  |  |  |  |  |  |
|  | FOLFIRI |  |  |  |  |  |  |  |  |  |
|  | Substance P | Low | 1.00 |  |  | 0.648 | 1.00 |  |  | 0.464 |
|  |  | High | 1.37 | 0.35 | 5.33 |  | 0.46 | 0.06 | 3.69 |  |
|  | FOLFOX |  |  |  |  |  |  |  |  |  |
|  | Substance P | Low | 1.00 |  |  | 0.214 | 1.00 |  |  | 0.919 |
|  |  | High | 3.67 | 0.47 | 28.40 |  | 1.11 | 0.15 | 8.37 |  |
|  | XELOX |  |  |  |  |  |  |  |  |  |
|  | Substance P | Low | 1.00 |  |  | 0.028 | NA |  |  |  |
|  |  | High | 15.00 | 1.34 | 167.64 |  |  |  |  |  |
| Abbreviation: NA, not applicable | |  |  |  |  |  |  |  |  |  |
